# Attenuation of Tumor Burden in Response to Rucaparib in Lung Adenocarcinoma: The Contribution of Oxidative Stress, Apoptosis, and DNA Damage

**DOI:** 10.3390/ijms24032580

**Published:** 2023-01-30

**Authors:** Maria Pérez-Peiró, Paula Valentí-Serra, Blanca León-González, Coral Ampurdanés, Xavier Duran, José Yélamos, Esther Barreiro

**Affiliations:** 1 Pulmonology Department-Muscle Wasting and Cachexia in Chronic Respiratory Diseases and Lung Cancer Research Group, IMIM-Hospital del Mar, Parc de Salut Mar, Health and Experimental Sciences Department (CEXS), Universitat Pompeu Fabra (UPF), Barcelona Biomedical Research Park (PRBB), 08003 Barcelona, Spain; 2 Centro de Investigación en Red de Enfermedades Respiratorias (CIBERES), Instituto de Salud Carlos III (ISCIII), 08003 Barcelona, Spain; 3 Cancer Research Program, Hospital del Mar Medical Research Institute (IMIM)—Hospital del Mar, 08003 Barcelona, Spain; 4 Scientific, Statistics, and Technical Department, Hospital del Mar—IMIM, Parc de Salut Mar, 08003 Barcelona, Spain

**Keywords:** lung adenocarcinoma experimental model, PARP inhibitor rucaparib, DNA damage and apoptosis, cell proliferation, PARP activity, protein oxidation, antioxidants

## Abstract

In cancer, overactivation of poly (ADPribose) polymerases (PARP) plays a relevant role in DNA repair. We hypothesized that treatment with the PARP inhibitor rucaparib may reduce tumor burden via several biological mechanisms (apoptosis and oxidative stress) in mice. In lung tumors (LP07 lung adenocarcinoma) of mice treated/non-treated (control animals) with PARP inhibitor (rucaparib,150 mg/kg body weight/24 h for 20 day), PARP activity and expression, DNA damage, apoptotic nuclei, cell proliferation, and redox balance were measured using immunoblotting and immunohistochemistry. In lung tumors of rucaparib-treated mice compared to non-treated animals, tumor burden, PARP activity, and cell proliferation decreased, while DNA damage, TUNEL-positive nuclei, protein oxidation, and superoxide dismutase content (SOD)2 increased. In this experiment on lung adenocarcinoma, the pharmacological PARP inhibitor rucaparib elicited a significant improvement in tumor size, probably through a reduction in cell proliferation as a result of a rise in DNA damage and apoptosis. Oxidative stress and SOD2 also increased in response to treatment with rucaparib within the tumor cells of the treated mice. These results put the line forward to the contribution of PARP inhibitors to reduced tumor burden in lung adenocarcinoma. The potential implications of these findings should be tested in clinical settings of patients with lung tumors.

## 1. Introduction

Non-small cell lung cancer (NSCLC) continues to be the leading cause of death attributable to cancer in many countries [1,2,3,4]. In these patients, surgical resection of the lung tumors is the elective therapy [5,6,7]. Nonetheless, many patients are not suitable for surgical treatment due to the advanced stage of the disease and/or their impaired health status. Chemotherapy and immune therapy along with other biological agents can be prescribed in those specific scenarios [3,8,9].

Poly ADP-ribosylation (PARylation) forms poly (ADP-ribose, PAR) polymers that vary in size and branch within cells. Functional and structural changes on the target proteins take place as a result of the action of attached PAR polymers [10]. In order to maintain DNA stability, integrity, and repair, overactivation of PARP occurs in response to DNA damage [11]. PARP-1 and PARP-2 play a critical role in DNA repair, but they may also promote carcinogenesis and tumor progression in tissues [12,13,14,15,16,17]. In this regard, PARP-1 overexpression correlated with poor survival in breast cancer patients [18].

In cancer treatment, the inhibition of PARP activity represents a promising tool. PARP activity inhibition fosters the action of chemotherapy, immune therapy, and radiotherapy [19]. In tumors characterized by BRCA mutations, PARP inhibitors may also be administered alone such as in breast and ovarian cancer types [20]. Moreover, in a preclinical model of SCLC, the combination of platinum based-chemotherapy with PARP inhibitors showed a better efficacy than chemotherapy alone [21]. Importantly, in another preclinical model of NSCLC in which *Parp*-1^−/−^ and *Parp*-2^−/−^ mice were studied, tumor burden was significantly reduced through several mechanisms such as increased oxidative stress, apoptosis, and autophagy compared to wild type animals [22]. 

Pharmacological inhibitors of PARP-1 and -2 have also proven to be beneficial in the treatment of other cancer types such as LC in patients [12,13,14,15,16,23,24,25,26,27]. Furthermore, the association of different PARP inhibitors such as niraparib and olaparib with cisplatin also induced additive effects on the treatment of LC and in other cancer types, namely cervical, liver, and testicular cancer [23,24,26,27,28]. The inhibitor rucaparib is also currently being used in clinical settings for the treatment of recurrent ovarian, fallopian tube, and primary peritoneal cancer.

The precise mechanisms whereby PARP-1 and -2 inhibitors may reduce tumor size and growth remain to be fully understood. Redox imbalance underlies the pathophysiology of many different acute and chronic conditions including LC [29,30,31,32]. Increased levels of reactive oxygen species (ROS), namely hydroxyl and superoxide anion, were shown in LC tumorigenesis [31,33]. Additionally, cell death [34] and autophagy [35,36] may also be signaled by a rise in oxidant production in cells. PARP-1 and PARP-2 enzymes are also activated in response to increased oxidative stress [37,38]. Apoptosis and necrosis may also be triggered by PARP-1 activity via enhanced oxidative stress [39,40]. In another investigation [22], a reduction in tumor burden was observed in mice that were genetically deficient for PARP-1 and PARP-2 expression and activity through increased oxidative stress.

A recent meta-analysis reveale45d no significant differences in terms of safety and tolerability with the use of the different pharmacological inhibitors currently being used in clinical settings [29]. Interestingly, the PARP inhibitor rucaparib was shown to exert beneficial effects on the respiratory and limb muscles of LC-induced cachectic mice through attenuation of muscle damage and structural abnormalities along with a significant impact on their physical activity [30]. Whether rucaparib may also induce a decrease in tumor burden through different biological mechanisms, including oxidative stress, remains to be fully elucidated.

Therefore, we hypothesized that treatment with the PARP inhibitor rucaparib may reduce tumor burden via several biological mechanisms in mice. Accordingly, the main objectives in the current investigation were to assess, in lung adenocarcinoma tumors of BALB/c mice treated with rucaparib, the following parameters: (1) PARP activity levels, (2) PARP-1 and PARP-2 protein expression, (3) DNA damage, (4) cell proliferation, (5) protein oxidation levels, and (6) antioxidants. Non-treated LC mice were also used as the control group.

## 2. Results

### 2.1. Physiological and Tumor Characteristics in LC Mice

In tumor-bearing mice + rucaparib, the tumor weight and area were significantly lower (33% and 31%, respectively) than in non-treated tumor-bearing mice (Table 1). The body weight gain significantly improved in tumor-bearing mice + rucaparib compared to non-treated animals (Table 1). Other parameters such as initial and final body weight with and without the tumor did not significantly differ between the two study groups (Table 1). In both experimental groups, tumor weight was inversely correlated with the final body weight gain with and without the tumor (Table 2).

### 2.2. PARP Activity Decreased in Tumors Treated with Rucaparib

**Figure 1:**
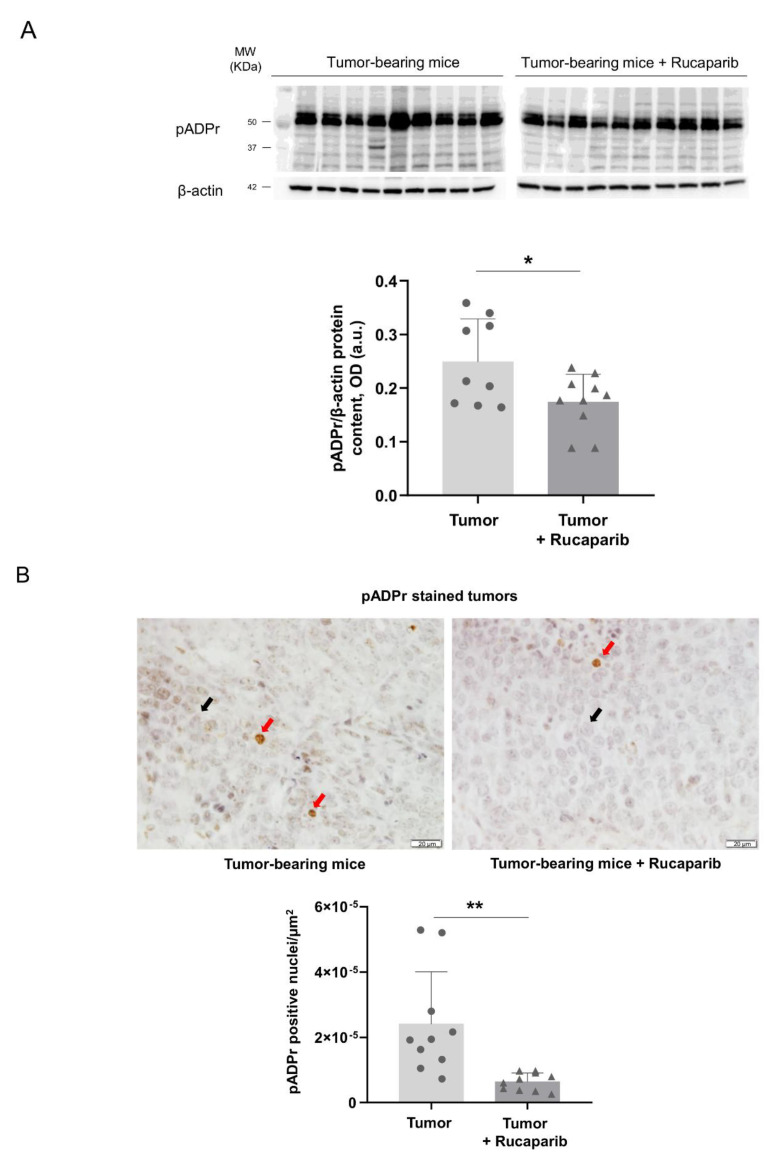
(**A**) Representative immunoblots of pADPr polymers and β-actin proteins in the tumors of both study groups (**top**) and mean values and standard deviations of pADPr protein content (**bottom**). (**B**) Representative examples of nuclei positively stained for poly-ADPr (red arrows point towards brown nuclei) and not stained (black arrows point towards blue nuclei, hematoxylin counterstaining) in tumors of both experimental groups (**top**) and mean values and standard deviations of positively stained nuclei for pADPr in the total measured area (**bottom**). In the scatter plot, the circles represent the untreated tumor-bearing mice while the triangles represent the rucaparib treated tumor-bearing mice. Statistical significance: * *p* ≤ 0.05, ** *p* ≤ 0.01 between tumor-bearing mice treated with rucaparib and non-treated tumor-bearing mice. Definition of abbreviations: pADPr, poly-ADP ribose; OD, optical densities; a.u., arbitrary units. Scale bar = 20 μm, 40× magnification.

**Figure 2:**
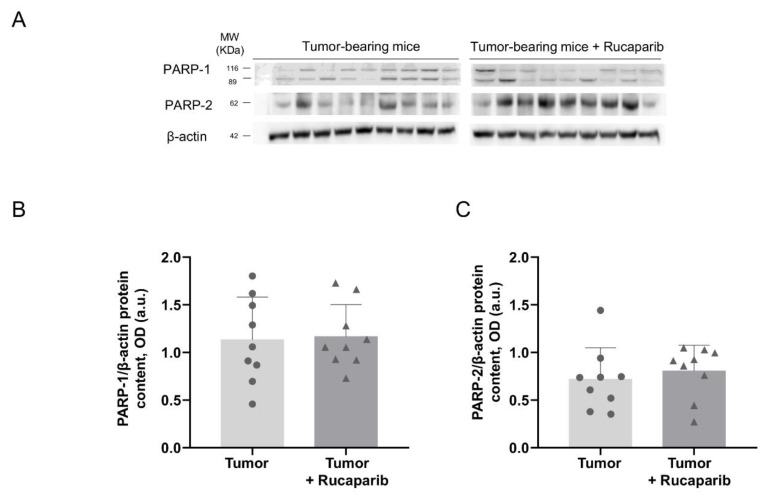
Representative immunoblots of PARP-1, PARP-2 and β-actin proteins in the tumors of both study groups (**A**). Mean values and standard deviations of PARP-1 (**B**) and PARP-2 (**C**) protein content. In the scatter plot, the circles represent the untreated tumor-bearing mice while the triangles represent the rucaparib treated tumor-bearing mice. Definition of abbreviations: PARP, poly-ADP ribose polymerase; OD, optical densities; a.u., arbitrary units.

The PARP activity levels as measured using two different techniques were significantly lower in cancer specimens of tumor-bearing mice treated with rucaparib than in non-treated animals (33% and 71%, respectively, Figure 1A,B). No significant differences in the levels of PARP-1 and PARP-2 protein expression were found in tumors between the two experimental groups (Figure 2A,B).

### 2.3. DNA Damage Increased in Tumors Treated with Rucaparib

**Figure 3:**
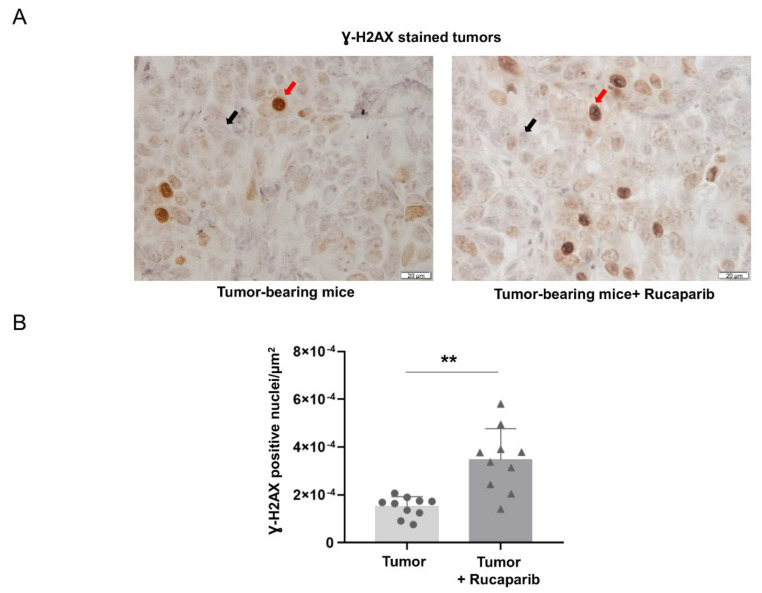
Representative examples of nuclei positively stained for Ɣ-H2AX (red arrows point towards brown nuclei) and not stained (black arrows point towards blue nuclei, hematoxylin counterstaining) in tumors of both experimental groups (**A**). Mean values and standard deviations of positively stained nuclei for Ɣ-H2AX in the total measured area (**B**). In the scatter plot, the circles represent the untreated tumor-bearing mice while the triangles represent the rucaparib treated tumor-bearing mice. Statistical significance: ** *p* ≤ 0.01 between tumor-bearing mice treated with rucaparib and non-treated tumor-bearing mice. Definition of abbreviations: Ɣ-H2AX, H2A histone family member X. Scale bar = 20 μm, 40× magnification.

The DNA damage as measured using the marker γ-H2AX within the cells was significantly higher (133%) in cancer specimens of tumor-bearing mice treated with rucaparib than in non-treated animals (Figure 3A,B).

### 2.4. TUNEL-Positive Nuclei in Tumors Treated with Rucaparib

**Figure 4:**
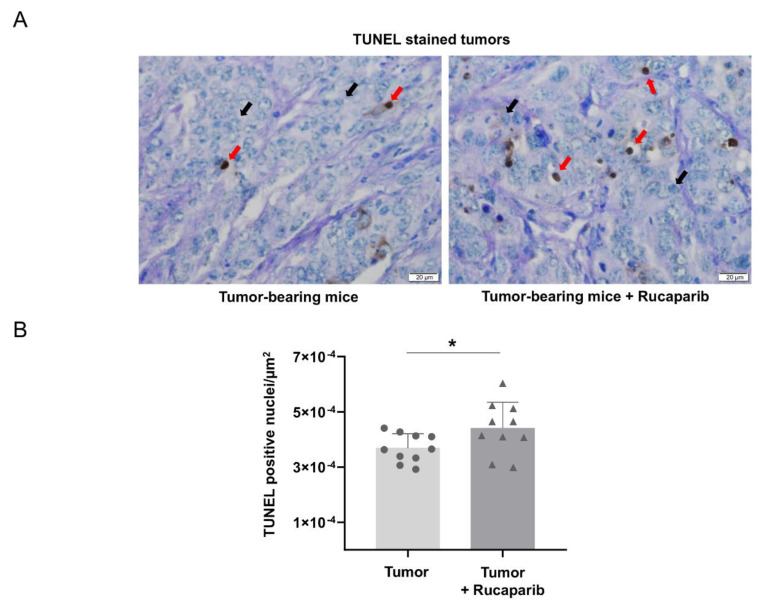
Representative examples of nuclei positively stained for TUNEL (red arrows point towards brown nuclei) and not stained (black arrows point towards blue nuclei, methyl green counterstaining) in the tumors of both experimental groups (**A**). Mean values and standard deviations of positively stained nuclei for TUNEL in the total measured area (**B**). In the scatter plot, the circles represent the untreated tumor-bearing mice while the triangles represent the rucaparib treated tumor-bearing mice. Statistical significance: * *p* ≤ 0.05 between tumor-bearing mice treated with rucaparib and non-treated tumor-bearing mice. Definition of abbreviations: TUNEL, Terminal deoxynucleotidyl transferase dUTP nick end labelling. Scale bar = 20 μm, 40× magnification.

The TUNEL-positive nuclei counts significantly increased (20%) in the specimens of tumor-bearing mice treated with rucaparib compared to the non-treated animals (Figure 4A,B).

### 2.5. Cellular Proliferation Decreased in Tumors Treated with Rucaparib

**Figure 5:**
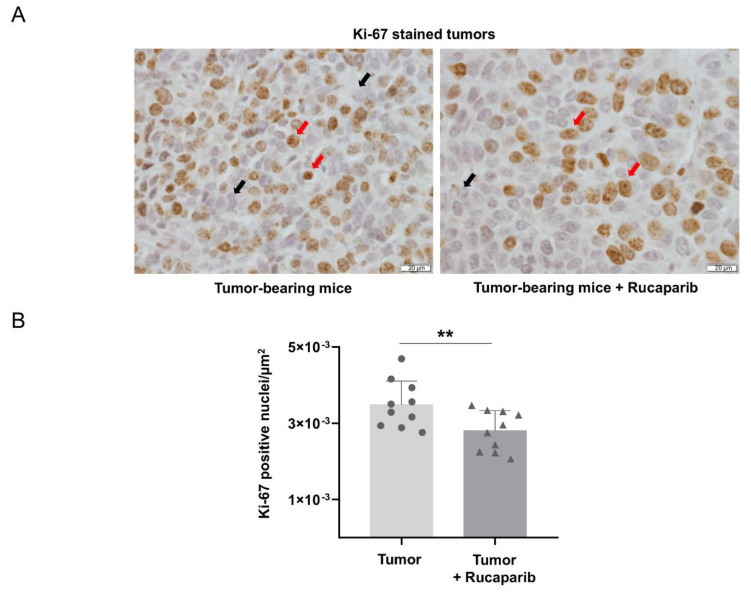
Representative examples of nuclei positively stained for Ki-67 (red arrows point towards brown nuclei) and not stained (black arrows point towards blue nuclei, hematoxylin counterstaining) in tumors of both experimental groups (**A**). Mean values and standard deviations of positively stained nuclei for Ki-67 in the total measured area (**B**). In the scatter plot, the circles represent the untreated tumor-bearing mice while the triangles represent the rucaparib treated tumor-bearing mice. Statistical significance: ** *p* ≤ 0.01 between tumor-bearing mice treated with rucaparib and non-treated tumor-bearing mice. Scale bar = 20 μm, 40× magnification.

The levels of Ki-67 positive cells were significantly lower (28%) in the cancer specimens of tumor-bearing mice treated with rucaparib than in non-treated animals (Figure 5A,B). An almost significant inverse relationship was observed between Ki-67 positive cells and PARP activity in tumors of non-treated mice, while a significant positive correlation was observed between those variables in tumors of the treated animals (Table 2).

### 2.6. Redox Balance in Tumors in Response to Rucaparib Treatment

**Figure 6:**
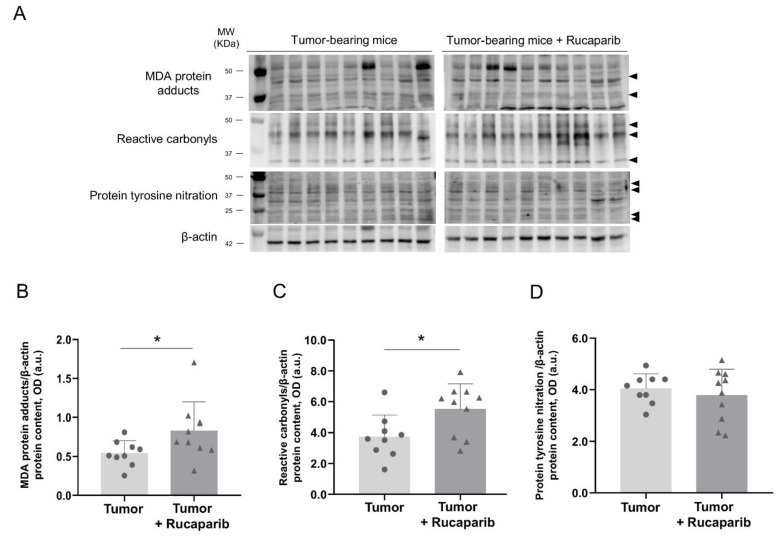
Representative immunoblots of MDA-protein adducts, protein tyrosine nitration, reactive carbonyls and β-actin proteins in the tumors of both study groups (**A**). Mean values and standard deviations of MDA protein adducts (**B**), protein tyrosine nitration (**C**) and reactive carbonyls (**D**) protein content. In the scatter plot, the circles represent the untreated tumor-bearing mice while the triangles represent the rucaparib treated tumor-bearing mice. Statistical significance: * *p* ≤ 0.05 between tumor-bearing mice treated with rucaparib and non-treated tumor-bearing mice. Definition of abbreviations: MDA, malondialdehyde; OD, optical densities; a.u., arbitrary units.

Oxidative stress markers. The protein carbonylation as measured by levels of MDA-protein adducts and reactive carbonyls were significantly greater (59% and 44%, respectively) in the tumors of the animals treated with rucaparib than in those of non-treated mice (Figure 6A–C). The levels of MDA-protein adducts positively correlated with the tumor weight in both experimental groups of mice (Table 2).

The tumor protein nitration levels, however, did not significantly differ between the two groups (Figure 6A,D). A significant correlation between protein tyrosine nitration levels and tumor weight was found in the animals treated with rucaparib, while such a correlation was not seen in the non-treated animals (Table 2).

**Figure 7:**
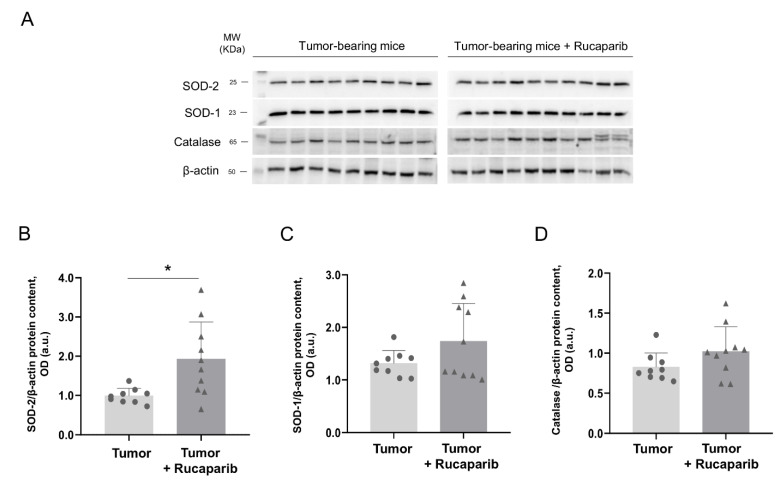
Representative immunoblots of SOD-1, SOD-2, catalase and β-actin proteins in the tumors of both study groups (**A**). Mean values and standard deviations of SOD-1 (**B**), SOD-2 (**C**) and catalase (**D**) protein content. In the scatter plot, the circles represent the untreated tumor-bearing mice while the triangles represent the rucaparib treated tumor-bearing mice. Statistical significance: * *p* ≤ 0.05 between tumor-bearing mice treated with rucaparib and non-treated tumor-bearing mice. Definition of abbreviations: SOD, superoxide dismutase; OD, optical densities; a.u., arbitrary units.

The protein levels of Mn-SOD significantly increased (96%) in tumor-bearing mice treated with rucaparib compared to non-treated animals (Figure 7A,B). Nonetheless, no significant differences were detected in the protein expression of SOD1 or catalase in the tumors between the two study groups (Figure 7A,C,D).

## 3. Discussion

In the present study, the most relevant novel findings were that treatment with the pharmacological PARP inhibitor rucaparib elicited a significant reduction in tumor size (~30%) along with an improvement in body weight gain in the mice compared to the non-treated animals. Furthermore, in the rucaparib-treated mice, a decrease in PARP activity was also observed, while no differences in PARP-1 or PARP-2 expression were detected between the two groups. Moreover, the levels of DNA damage and TUNEL-positively stained nuclei increased in the tumors of the mice treated with the PARP inhibitor, whereas the cell proliferation marker Ki-67 decreased in those tumors. Finally, a rise in protein oxidation markers was also seen in the tumors of the mice treated with rucaparib along with an increase in the powerful antioxidant SOD2. Significant correlations were observed between the levels of MDA-protein adducts and tumor weight in the mice treated with rucaparib. Additionally, in both experimental groups, significant inverse correlations were observed between the tumor weight and total body weight gain with and without the tumor, confirming the validity and reliability of the experimental model. As far as we are concerned, these are all novel findings that push the line forward on the role played by the PARP inhibitor rucaparib in tumor burdens in an experimental mode of LC.

The pharmacological inhibition of PARP was demonstrated in the tumors of the mice treated with rucaparib, as a significant decline in PARP activity was detected using two different laboratory approaches. Rucaparib is a well-known inhibitor of PARP-1, PARP-2, and PARP-3 enzymes that is clinically used for the treatment of certain types of ovarian tumors [31]. In the present study, the inhibition of PARP activity was attained in the adenocarcinoma tumors of the mice that received treatment with rucaparib. These results are also in agreement with previous investigations in which pharmacological PARP inhibitors elicited a decline in PARP activity in several experimental models [30,32,33]. In line with previous results [32,34], the expression of either PARP-1 or PARP-2 molecules in the tumors did not significantly differ between the two study groups. The attenuation in PARP activity was not dependent on the content of the two major PARP isoforms in this experimental model.

Interestingly, the safety profile of the four approved PARP inhibitors (olaparib, rucaparib, niraparib, and talazoparib) was comparable in terms of adverse events associated with the discontinuation of treatment [29]. On this basis, it could be argued that similar effects on tumor size and redox balance might be elicited by any of the PARP inhibitors that are currently used for the treatment of several cancer types in clinical settings. The design of future studies will be required to demonstrate those beneficial effects. In the present investigation, rucaparib was partly chosen due to its reported therapeutic potential in patients with NSCLC [29].

Histone H2AX is critical in the regulation of DNA damage [35], which in turn may be a mediator of increased apoptosis in several cancer types as a result of pharmacological PARP inhibition with and without chemotherapy [35]. As previously shown [34,36], a rise in DNA damage was also observed in the tumors of the mice treated with rucaparib compared to the non-treated animals, suggesting that the inhibition of PARP activity elicited such a rise in damaged DNA levels within the tumor cells. Furthermore, the treatment with rucaparib also elicited an increase in the number of TUNEL-positively stained nuclei in the mouse tumors. Clearly, these findings may account for the reduced tumor burden observed in the mice treated with rucaparib. Moreover, they are also in line with the results reported in previous investigations, in which increased apoptosis was also demonstrated in different tumor types, including lung cancer cells that were exposed to treatment with PARP inhibitors [41,42,43,44]. Importantly, a positive association between PARP activity and Ki-67 positive cells was observed in the tumors of mice treated with rucaparib, while such a relationship was negative in the tumors of the non-treated mice. These findings suggest that PARP activity influenced tumor cell proliferation, particularly in the animals treated with the inhibitor.

Interestingly, tumor cell proliferation as quantified by the Ki-67 marker was reduced in the adenocarcinoma cells of the mice treated with the PARP inhibitor rucaparib. These are consistent results with previous investigations [36,45,46,47], in which DNA damage was probably the major driver of the attenuation of tumor cell proliferation. 

In the present study, the levels of protein oxidation were significantly increased in the tumors of the mice treated with the PARP inhibitor rucaparib. These results are in line with those previously reported in which the absence of PARP-1 and PARP-2 expression also led to increased oxidative stress in the tumors of *Parp*-1^−/−^ and *Parp*-2^−/−^ knockout mice [22,48]. Furthermore, significant positive correlations were observed between the levels of oxidative stress markers and tumor weight in the animals treated with rucaparib. Oxidative stress mediates mechanisms of cell death, which may have accounted for the improvement observed in the tumor size and body weight gain in the mice treated with rucaparib [22,48]. In addition, the protein levels of the mitochondrial SOD were significantly increased in the tumors of the mice treated with rucaparib, whereas no differences were seen in cytosolic SOD or catalase protein levels. These results are partly in agreement with those previously reported in several experimental models [36,46,49,50]. These findings suggest that SOD2 may be used as a marker of response to the treatment with PARP pharmacological inhibitors.

### Study Limitations

A potential limitation might be related to the animal experimental model and to what extent these results can be applied to clinical settings. A second limitation may be related to the duration of the beneficial effects observed in the tumors of the mice in the medium- and long-term. As occurs in clinical settings with patients, maintenance of the effects detected in the tumors will require the administration of the pharmacological inhibitor on a continuous basis. The potential side-effects that may take place in other organs as a result of the systemic administration of the compound may also warrant attention in future studies. Whether other PARP inhibitors that are currently in use in clinical settings (namely olaparib, niraparib, or talazoparib) may also induce similar effects on the tumors should be explored in future investigations. We believe that the results obtained in the present study can serve as the basis to better understand the mechanisms whereby PARP may be involved in the pathophysiology of lung tumorigenesis. The elucidation of these mechanisms may also help identify additional molecular pathways with a potential to be therapeutically targeted in future investigations.

## 4. Materials and Methods

### 4.1. Experimental Model and Design

The in vivo experiments were carried out in the animal facilities at Barcelona Biomedical Research Park (PRBB) under specific pathogen-free conditions. This controlled study was designed in accordance with the ethical standards on animal experimentation (EU 2010/63 CEE, *Real Decreto* 53/2013 BOE 34, Spain) at PRBB and the Helsinki convention for the use and care of animals. Ethical approval was obtained by the Animal Research Committee (Animal welfare department, Catalonia, EBP-17-0005).

The LP07 murine cell line was derived from a P07 lung tumor developed spontaneously in the lung of a BALB/c mouse [37,38,39,40,51]. The LP07 cell line was generously provided by Dr. Urtreger, Dr. Diament, and Dr. Bal de Kier Joffé (Research Area Institute of Oncology “Angel H. Roffo”, Buenos Aires, Argentina). LP07 cells were maintained at 37 °C in a humidified, 5% CO2–air atmosphere in minimal essential media (MEM, Biowest, Nuaille, France) supplemented with 10% fetal bovine serum (FBS) (Thermo Fisher Scientific Inc., Waltham, MA, USA) and 1% of penicillin/streptomycin/fungizone solution: 10,000 U/mL penicillin, 10,000 µg/mL streptomycin, and 25 µg/mL fungizone (Thermo Fisher Scientific Inc.). Subcultures were performed using trypsin-ethylenediaminetetraacetic acid (EDTA, Sigma-Aldrich, Burlington, MA, USA) 1X in phosphate buffered saline (PBS, Thermo Fisher Scientific Inc.). The cells were expanded for three weeks in order to reach the required amounts of cells to be inoculated to the mice. Overall, 4 × 10^5^ LP07 cells, resuspended in 0.2 mL MEM, were injected subcutaneously into the left flank of the mice on day 0 [38,39,40,45,52].

**Figure 8:**
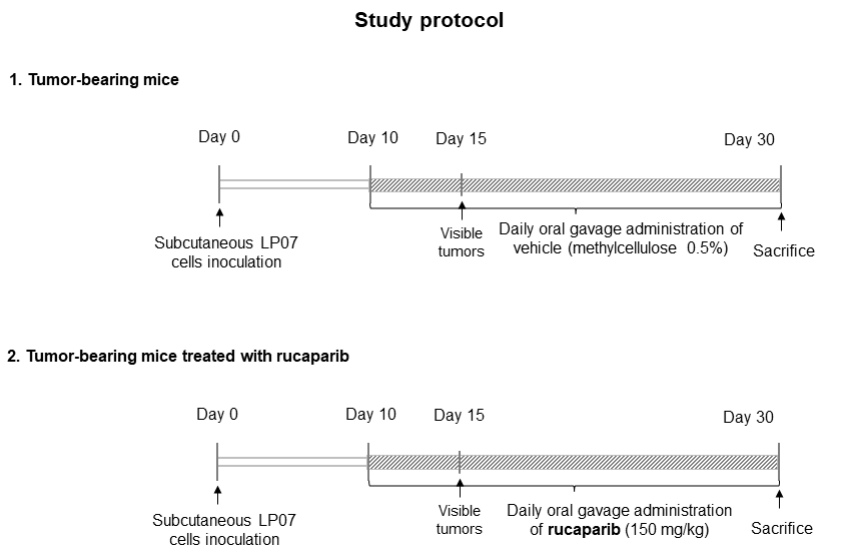
Schematic timeline representation of the study protocol in the two study groups.

Rucaparib was gently provided by Clovis Oncology (San Francisco, CA, USA) for the purpose of this study. A rucaparib solution was prepared following the instructions provided by Clovis Oncology. The dose of rucaparib was chosen on the basis of the recommendations provided by the European Medicines Agency (Procedure # EMEA/H/C/004272/0000) and those of Clovis. The treatment with rucaparib (150 mg/kg, dissolved in 0.5% methylcellulose) started on day 10, a time at which the tumors were visible, and was followed up until day 30 (Figure 8). The animals were treated daily (gavage procedures using a specific 20 G reusable feeding needle (Fine science tools Inc., Foster City, CA, USA) using a fresh-daily rucaparib solution). The control non-treated mice (sham controls) were administered a solution of 0.5% methylcellulose dissolved in distilled water (gavage) daily from day 10 up until day 30. All the study mice were sacrificed on day 30. 

Twenty BALB/c ten-week-old female mice (*n* = 10/group) were obtained from Harlan Interfauna Ibérica SL (Barcelona, Spain). The animals were divided into two different groups: (1) tumor-bearing mice group, treated daily with 0.5% methylcellulose, inoculation of LP07 cells resuspended in 0.2 mL MEM in the left flank, and (2) tumor-bearing mice group treated with 150 mg/kg/24 h rucaparib, inoculation of LP07 cells resuspended in 0.2 mL MEM in the left flank. The tumors were visible from day 14 thereafter up to the end of the study protocol (30 days).

### 4.2. Sacrifice and Sample Collection

All the animals were sacrificed on day 30 using an intraperitoneal injection of 0.1 mL sodium pentobarbital (60 mg/Kg) to induce anesthetic death. The confirmation of death in the euthanized animals was verified by evaluating the pedal and blink reflexes. The tumors were obtained from all of the mice. A fragment of the tumor was immediately frozen in liquid nitrogen and was preserved at −80 °C for further molecular analysis. Additionally, the remaining specimen of the tumor was immersed in an alcohol-formol bath to be thereafter embedded in paraffin as previously described [38,40,45,53].

### 4.3. In Vivo Measurements in the Mice

During the study period, food and water were supplied ad libitum to all the animals. The body weight and food intake were measured every 24 h. The tumor area was determined using a caliper and the formula: (L × W^2^)/2, where L is tumor length and W is tumor width. The tumor weight was determined using a scale at the end of the study.

### 4.4. Biological Analysis

*Immunoblotting.* Frozen tumor samples of all experimental mice groups were homogenized at 4 °C using a specific lysis buffer containing 50 mM 4-(2-hydroxyethyl)-1-piperazineethanesulfonic acid (HEPES, Sigma-Aldrich), 150 mM NaCl (Sigma-Aldrich), 100 nM NaF (Sigma-Aldrich), 10 mM Na pyrophosphate (Sigma-Aldrich), 5 mM EDTA, 0.5% Triton-X (Sigma-Aldrich), 2 μg/mL leupeptin (Sigma-Aldrich), 100 μg/mL phenylmethylsulfonyl fluoride (PMSF) (Sigma-Aldrich), 2 μg/mL aprotinin (Sigma-Aldrich), and 10 μg/mL pepstatin A (Sigma-Aldrich).

Protein samples (ranging from 20 to 50 μg, according to antigen and antibody) were diluted with an equal volume of 2X laemmli buffer (Bio-Rad Laboratories, Inc., Hercules, CA, USA) and 10% of 2-mercaptoethanol (Bio-Rad Laboratories). Afterwards, the samples were boiled for 5 min at 95 °C and were separated by electrophoresis. Then, the proteins were transferred onto polyvinylidene difluoride (PVDF) membranes, blocked with bovine serum albumin (BSA, NZYtech, Lisboa, Portugal) or with 5% nonfat milk and incubated with primary antibodies overnight at 4 °C. The primary antibodies used to analyze the protein content of the target biomarkers are described in Table 3.

Antigens from all the samples were detected using HRP-conjugated secondary antibodies (Jackson ImmunoResearch Inc., West Grove, PA, USA) and a chemiluminescence kit (Thermo Fisher Scientific). PVDF membranes from the different groups were scanned at the same time under identical exposure conditions by Alliance Q9 Advanced (Uvitec Cambridge, UK). The optical densities of specific bands were quantified using the ImageJ software (National Institute of Health, available at http://rsb.info.nih.gov/ij/, accessed on 1 July 2022). The membranes were stripped of primary and secondary antibodies through incubation with a stripping solution (25 nM glycine, pH 2.0, and 1% sodium dodecyl sulfate, SDS) (Sigma-Aldrich) for 30 min according to previously published methodologies [38,39,40,56,57] to detect the protein loading control β-actin for each of the markers. The optical densities obtained from each study marker were normalized to those of the loading control (β-actin). Negative controls were performed to detect non-specific bands on the blot, incubating the membranes with only a secondary antibody, for the resulting multiple bands (pADPr, MDA-protein adducts, reactive carbonyls, and protein tyrosine nitration). 

#### 4.4.1. Histological Analyses of Tumor Samples

PARP activity, DNA damage levels and cell proliferation were assessed in the tumors of both experimental groups by quantification of pADPr polymers, γ-H2AX (the phosphorylated form of H2AX), and Ki-67, respectively, using conventional immunohistochemistry as described in previous studies [10,22,58]. Briefly, tumor paraffin-embedded sections (three micrometers) were deparaffinized with xylene and rehydrated through a graded ethanol series. Antigen retrieval was performed with a 0.1 M citrate buffer (pH 6) in a pressure-cooker for 15 min (pADPr, γ-H2AX) or with a 1 mM ethylenediaminetetraacetic acid (EDTA) buffer and 0.05% of Tween20 (pH 8.0) at 95 °C for 40 min (Ki-67). After cooling for 30 min, endogenous peroxidase activity was blocked with 6% of H_2_O_2_. The sections were then rinsed in PBS and were subsequently incubated with a primary antibody at room temperature for 40 min (pADPr, γ-H2AX) or overnight at 4 °C (Ki-67) with the following specific primary antibodies: anti-pADPr (Santacruz biotechnology), anti-γ-Histone H2AX primary antibody (Merck-Millipore) and anti-Ki67 (Merck-Millipore). After primary antibody incubation, the sections were again rinsed with PBS three additional times. Slides were incubated with horseradish peroxidase (HRP) Polymer-antiMouse/Rabbit IgG (Neobiotech, Seoul, Republic of Korea) at room temperature for 30 min. After three washes with PBS, a 3,3′-Diaminobenzidine (DAB) solution (Neobiotech) was applied until the appropriate color (brown) was reached. The samples were rinsed under tap water for 10 min. The slides were counter stained with hematoxylin. The sections were dehydrated with an alcohol–xylene battery and were mounted in dibutylphthalate polystyrene xylene (DPX) media [52,59]. Negative control samples (PBS-only incubations) were run in each assay. Images of the stained sections were captured using a light microscope (×40 objective, Olympus, Series BX50F3, Olympus Optical Co., Hamburg, Germany). Positive stained nuclei for pADPr, γ-H2AX and Ki-67 were counted in all tumor sections and the tumor area was calculated using ImageJ software (National Institute of Health, available at http://rsb.info.nih.gov/ij/, accessed on 1 July 2022). Data are presented as the count of the pADPr polymers, γ-H2AX or Ki-67 positively stained cells in the measured area of the stained tumor sections.

#### 4.4.2. Terminal Deoxynucleotidyl Transferase-Mediated dUTP Nick-End Labeling (TUNEL) Assay

Apoptotic myonuclei were determined in 3-μm paraffin-embedded sections of tumor specimens using the TUNEL assay (ApopTag Peroxidase In Situ Apoptosis Detection Kit; Merck Millipore Darmstadt, Germany) as previously reported [49,52]. In brief, DNA strand breaks that are generated during nuclear activation can be identified by labeling the 3′-OH terminal groups. The labeling of the 3′-OH groups with modified nucleotides is carried out by an enzymatic reaction catalyzed by the terminal deoxynucleotidyl transferase (TdT) enzyme. Tumor sections were fixed, permeabilized, and immediately incubated with the TUNEL Working Strength TdT Enzyme and the anti-Digoxigenin Conjugate. TdT catalyzed the addition of digoxigenin-dNTP at 3′-OH terminal groups in single- and double-stranded DNA. After washing, the digoxigenin-nucleotides that were bound to DNA fragments were detected using an anti-digoxigenin antibody conjugated with peroxidase, which resulted in a brown color upon reaction. A methyl green counterstain was also used to distinguish the negative staining nuclei. Negative control experiments, in which the TdT enzyme was not added, were also performed. The final count of positive and total nuclei was carried out by two trained observers (correlation coefficient 95%). The apoptotic nuclei were expressed as the percentage of the TUNEL-positive nuclei to the total number of counted nuclei [49,52].

### 4.5. Statistical Analysis

In the investigation, the normality of all the variables was assessed using the Shapiro–Wilk test. Accepting an alpha risk of 0.05 and a beta risk of 0.2 (80% statistical power) in a two-sided test, a minimum of nine mice was necessary in each group to recognize a minimum difference of 0.54 units in the mean value of the tumor weight variable to be considered as statistically significant. The standard deviation was assumed to be 0.4 for this variable. The differences between the two groups (untreated tumor-bearing mice and rucaparib treated tumor-bearing mice) were assessed using T-Student test for the parametric variables and Mann–Whitney U test for the non-parametric ones. The physiological and tumor characteristic variables are represented in a table, while biological variables obtained from the tumor specimens are shown in data point graphs. The potential associations between physiological and biological variables were assessed using the Pearson’s correlation coefficient. Such correlations were explored within each group of mice individually. The correlations were mainly targeted for the variables whose mean values showed a statistically significant difference between the two experimental groups. The statistical significance was established at *p* ≤ 0.05. The statistical analyses of the study were performed using the software Statistical Package for the Social Sciences (SPSS, version 23, SPSS Inc., Chicago, IL, USA).

## 5. Conclusions

The novel findings encountered in this study were that the pharmacological PARP inhibitor rucaparib elicited a significant improvement in tumor size, probably through a reduction in the cell proliferation rates resulting from the increased levels of DNA damage and apoptosis seen in the lung adenocarcinoma tumors of the treated mice. Oxidative stress markers and SOD2 also increased in response to treatment with rucaparib within the tumor cells of the treated animals. These results push the line forward to the contribution of PARP inhibitors to reduced tumor burden in lung adenocarcinoma through the action of three major biological mechanisms, namely DNA damage, oxidative stress, and apoptosis. The potential implications of these findings should be tested in the clinical settings of patients with lung tumors. Other PARP inhibitors that are also currently being used in the clinics may also elicit similar effects, which should be examined in future investigations.

## Figures and Tables

**Table 1:** Physiological and tumor characteristics in the study groups of mice.

Variables	Tumor- Bearing Mice	Tumor-Bearing Mice + Rucaparib
Tumor weight (g)	1.63 (0.33)	1.09 (0.40) **
Tumor area (mm^2^)	1755.06 (556.61)	1216.06 (444.03) *
Body weight gain (%)	−8.65 (4.66)	+2.10 (9.01) **
Body weight gain without tumor (%)	−12.96 (7.68)	−4.02 (11.21) *
Initial body weight (g)	20.86 (0.80)	20.43 (0.80)
Final body weight (g)	19.78 (1.68)	20.91 (2.11)
Final body weight without tumor (g)	18.27 (1.81)	19.57 (2.50)

Variables are presented as mean (standard deviation). Definition of abbreviations: g, gram; mm, millimeter. Statistical significance: * *p* ≤ 0.05, ** *p* ≤ 0.01 between tumor-bearing mice treated with rucaparib and non-treated tumor-bearing mice.

**Table 2:** Associations of the study variables analyzed in the tumors of the experimental groups.

	Body Weight Gain (%)	Body Weight Gain without Tumor (%)	MDA-Protein Adducts (a.u)	Protein Tyrosine Nitration (a.u)	Ki-67 (Positive Nuclei/µm^2^)
Tumor-bearing mice					
Tumor weight (g)					
r	−0.689	−0.764	0.895	−0.501	-
p	0.059	0.046	0.016	0.311	
pADPr (positive nuclei/µm^2^)					
r	-	-	-	-	−0.573
p					0.084
Tumor-bearing mice + Rucaparib					
Tumor weight (g)					
r	−0.671	−0.759	0.919	0.898	-
p	0.034	0.011	0.001	0.002	
pADPr (positive nuclei/µm^2^)					
r	-	-	-	-	0.580
p					0.048

**Table 3:** List of primary antibodies used in Western blot analysis.

Antibody	Dilution	Catalog Number	Supplier
Anti-pADPr antibody	1:500	sc-56198	Santa Cruz Biotechnology, Dallas, TX, USA
Anti-PARP-1 antibody	1:20	-	In-house generated affinity purified monoclonal antibody (clone A6.4.12) [54]
Anti-PARP-2 antibody	1:1000	-	In-house generated affinity purified monoclonal antibody (clone 4G8) [55]
Anti- MDA-protein adducts antibody	1:2000	MD20A-G1a	Academy Bio-Chemical Company, Houston, TX, USA)
Anti-3-nitrotyrosine antibody	1:1000	A-21285	Thermo Fisher Scientific, Waltham, MA, USA
Protein carbonyl assay kit	1:5000	Ab178020	Abcam, Cambridge, UK
Anti-SOD-1 antibody	1:2000	sc-17767	Santa Cruz Biotechnology
Anti-SOD-2 antibody	1:2000	sc-30080	Santa Cruz Biotechnology
Anti-catalase antibody	1:4000	219010	Merck KGaA, Darmstadt, Germany
Anti-β-actin, antibody	1:5000	sc-47778	Santa Cruz Biotechnology

Definition of abbreviations: pADPr, poly(ADP-ribose); PARP, poly(ADP-ribose)polymerase; MDA, malondialdehyde; SOD, superoxide dismutase.

## Data Availability

The datasets generated and analyzed during the current study are available from the corresponding author upon reasonable request.
